# Patterns of residual HIV-1 RNA shedding in the seminal plasma of patients on effective antiretroviral therapy

**DOI:** 10.1186/s12610-017-0063-x

**Published:** 2017-09-08

**Authors:** Christophe Pasquier, Marie Walschaerts, Stéphanie Raymond, Nathalie Moinard, Karine Saune, Myriam Daudin, Jacques Izopet, Louis Bujan

**Affiliations:** 1 INSERM U1043, CPTP, CHU Purpan, BP 3028, F-31024 Toulouse, France; 2 Université de Toulouse, UPS, CPTP, F-31024 Toulouse, France; 3 Laboratoire de Virologie, CHU de Toulouse, Hôpital Purpan, F-31059 Toulouse, France; 4 Université de Toulouse, UPS, Groupe de Recherche en Fertilité Humaine (EA 3694, Human Fertility Research Group), Toulouse, France; 5 CECOS Midi-Pyrénées, Hôpital Paule-de-Viguier, Toulouse, France; 6 Laboratoire de Virologie, Institut Fédératif de Biologie, 330 avenue de Grande Bretagne, TSA40031, 31059, Cédex 9 Toulouse, France

**Keywords:** HIV, Antiretroviral treatment, Sexual transmission, Semen, Sperm processing, Medically assisted reproduction, HIV, traitement antirétroviral, transmission sexuelle, sperme, préparation du sperme

## Abstract

**Background:**

More and more HIV-1-infected men on effective antiretroviral treatment (ART) have unprotected sex in order to procreate. The main factor influencing transmission is seminal HIV shedding. While the risk of HIV transmission is very low, it is difficult to assess in individuals. Nevertheless, it should be quantified.

**Results:**

We retrospectively analysed seminal plasma HIV-1 shedding by 362 treated HIV-infected men attending a medically assisted reproduction centre (1998–2013) in order to determine its frequency, the impact of the antiretroviral regimen on HIV shedding, and to identify shedding patterns. The HIV-1 virus loads in 1396 synchronized blood and semen samples were measured, and antiretroviral treatment, biological and epidemiological data were recorded.

We detected isolated HIV-1 shedding into the seminal plasma in 5.3% of patients on efficient antiretroviral treatment, but there was no association with the HIV antiretroviral drug regimen or the CD4 cell count. These men had undergone more regimen changes since treatment initiation and had been on the ongoing drug regimen longer than the non-shedding men. The patterns of HIV seminal shedding among patients with undetectable HIV blood virus load varied greatly. HIV seminal shedding can occur as long as 5 years after starting antiretroviral treatment.

**Conclusions:**

The seminal HIV load was used to monitor risk for infertile HIV-infected patients on an assisted reproductive technology program. This can still be recommended for patients who recently (6 months) started ART, or those with a poor history of adherence to ART but may also be usefull for some patients during counselling. Residual HIV seminal shedding is probably linked to breaks in adherence to antiretroviral treatment but local genital factors cannot be ruled out.

## Abstrait

**Résumé:**

De plus en plus d’hommes sous traitement antirétroviral (ART) ont des rapports sexuels non protégés à des fins de procréation. Le principal déterminant de la transmission sexuelle est. l’excrétion séminale du VIH. Malgré un risque de transmission très faible, il reste difficile à évaluer au niveau individuel. Dans ce contexte, l’étude de l’excrétion séminale du VIH, notamment chez des hommes sous traitement antirétroviral, est. d’un grand intérêt.

**Résultats:**

Nous avons analysé rétrospectivement l’excrétion séminale du HIV chez 362 hommes sous traitement antirétroviral consultant un centre d’assistance médicale à la procréation (1998–2013) pour déterminer sa fréquence, l’impact des antirétroviraux sur l’excrétion du HIV et identifier les profils d’excrétion. Les charges virales HIV-1 ont été mesurées dans 1396 échantillons de sang et de sperme prélevés concomitamment et les traitements, les données biologiques et épidémiologiques recueillis. Nous avons détecté une excrétion dans le plasma séminal isolée chez 5,3% des patients sous traitement antirétroviral efficace mais nous n’avons pas trouvé d’association avec la composition du traitement antirétroviral ou le taux de lymphocytes T CD4+. Ces hommes avaient eu plus de changements thérapeutiques et leur traitement avait été instauré depuis plus longtemps que pour les hommes non excréteurs. Les profils d’excrétion séminale du HIV parmi les patients avec une charge virale indétectable dans le sang étaient très variables. L’excrétion séminale du HIV peut survenir jusqu’à 5 ans après l’instauration du premier traitement antirétroviral.

**Conclusions:**

La charge virale séminale du HIV était l’outil classique d’évaluation du risque viral de transmission pour les patients infertiles infectés par HIV et inclus dans les programmes d’assistance médicale à la procréation. Ceci peut continuer à être recommandé chez les patients ayant débuté un traitement antirétroviral dans les 6 mois précédent ou chez ceux avec des antécédents de mauvaise adhérence au traitement mais peut aussi être utile pour le conseil de certains patients. Le risque résiduel d’excrétion séminale du HIV est. probablement lié à des défauts d’adhérence au traitement antirétroviral mais des facteurs génitaux ne peuvent pas être éliminés.

## Background

Anti-retroviral therapy (ART) has had a tremendous effect on HIV-1 replication. The HIV-1 RNA in the blood plasma of HIV-infected men becomes undetectable (NBVL: negative blood viral load) in less than 6 months and remains so for years. This sustained suppression of virus in the blood requires strict adherence to antiretroviral treatment [1]. The HIV-1 RNA load in the seminal compartment is also drastically reduced by ART, dropping to below the limit of detection in most cases [2, 3], and the risk of sexual HIV-1 transmission is reduced by up to 96% [4]. ART therefore prevents the sexual transmission of HIV within a population [4]. Nevertheless, patients are at greatest risk of HIV seminal shedding during the six months following treatment initiation because the amount of HIV RNA in the semen decreases more slowly than does the virus in the blood [5, 6]. As a result, the Swiss federal commission for HIV/AIDS [7] stated, in 2008, that an infected subject on ART for more than 6 months with no other sexual transmitted disease and with undetectable blood HIV RNA does not transmit HIV to his partner.

However, several studies have shown that HIV-1 RNA can be detected in the seminal plasma (PSVL: positive seminal viral load), or in the female genital tract, despite the patient being on effective ART for more than 6 months [3, 5, 8–13]. Many factors seem to be associated with HIV replication within the male genital tract. Symptomatic or asymptomatic sexually transmitted infections (STI) as well as CMV and HSV-2 shedding are all associated with HIV shedding [14]. Suboptimal antiretroviral drug concentrations within the genital tract, due to breaks in compliance, drug interactions or poor drug diffusion into the genital tract could also be involved [15]. Most HIV shedding in asymptomatic patients on effective ART with a low risk of STI and attending a medically assisted reproduction clinic, seems to be linked to the type of antiretroviral regimen and adherence to treatment: HIV shedding is reduced when ART effectiveness and tolerance are improved [3]. The shedding of HIV into the semen of men on ART may contribute to the residual risk of HIV transmission, but the size of this contribution is not known [16].

We therefore determined the frequency of men with isolated HIV seminal shedding (shedders). HIV RNA was detected in the seminal plasma of 362 HIV-infected men on ART who had an undetectable blood plasma HIV load (negative blood virus load + positive seminal virus load: NBVL + PSVL). The data obtained from samples collected over a 16-year period were used to identify the patterns of isolated HIV seminal shedding and to analyse the antiretroviral drug classes associated with the shedding.

## Methods

### Patients

A total of 1396 pairs of seminal plasma and blood samples were collected from 362 male patients between January 1998 and December 2013 and tested for HIV-1 RNA. All the men were HIV-1-infected partners of uninfected women who wanted to become pregnant using washed sperm and assisted reproduction [17]. They were attending Toulouse University medically assisted reproduction centre and were managed according to French law and guidelines. These specify clinical and biological inclusion criteria, spermatozoa processing and assisted reproductive technology as well as adherence to ethical issues and require an informed consent signature. Patient follow-up began in January 1998 and most men were already on antiretroviral treatment. The initial phase of the follow-up (1998–2003) was conducted using a French AIDS research agency protocol (ANRS 096) which had been approved by our review board (Comité de Protection des Personnes dans la Recherche Biomédicale Toulouse II) and the later phase (2003–2013) as set out in the French legislation covering the care of HIV serodiscordant couples wishing to have children. All subjects underwent a clinical andrological examination during their first visit to the centre, at which time their HIV history (HIV risk groups, age, time of HIV diagnosis, CD4 T cell count, hepatitis B (HBV) and hepatitis C (HCV) coinfections, ART regimens, time of ART initiation), genital-urinary infections and other disorders were recorded. Changes in antiretroviral regimens or the subject’s health were recorded at follow-up visits. Data on the first antiretroviral treatment and current regimen plus the antiretroviral drug regimen followed during episodes of isolated HIV seminal shedding (NBVL + PSVL) were recorded and used to calculate the total durations of treatment and the current regimen.

Two subjects were not included in the study because of their very divergent responses to ART, with persistent HIV shedding into the seminal plasma that continued for over 3 years (2006–2008 and 2007–2010 respectively). Details of both cases have been published [18, 19].

### HIV-1 RNA assays

The HIV-1 RNA in blood plasma was quantified using the Cobas Taqman HIV-1 assay (Roche Diagnostics, Meylan, France; detection limit = 20 copies/ml) and the HIV-1 RNA in the seminal plasma was measured using a previously described validated protocol (detection limit = 200 copies/ml) [20, 21]. Each man provided multiple samples of semen during follow-up. The blood plasma virus load and seminal plasma virus load were defined as detectable/positive (PBVL / PSVL) or undetectable/negative (NBVL/NSVL) with cut offs of 20 and 200 copies/ml, respectively.

We analysed the influence of antiretroviral treatment on HIV shedding by comparing the shedders (at least one NBVL + PSVL, *n* = 22) with controls (controlled HIV replication in both compartments: NBVL + NSVL (*n* = 171). Untreated patients (*n* = 15) were excluded.

### Statistical analysis

Quantitative data for groups of patients were compared using the non-parametric Mann-Whitney U-test and categorical data by the Chi^2^ or Fisher’s exact tests. We used a generalized linear model to estimate trends and confidence intervals of the prevalence of shedders. Statistical analyses were performed using SAS software (version 9.3, SAS Institute, Inc.) and significance is defined as 5%.

## Results

### Patient characteristics

The mean age of the patients (*n* = 362) at the first consultation was 39 ± 6 (median = 39). Of these, 92% were treated during the study period, 83% (299/362) with a triple or more antiretroviral drug combination for a mean period of 7 ± 5 years (median = 7). The mean duration of HIV infection was 11 ± 6 years (median = 11) and the duration of follow-up between the first and last samples collected at our centre was 13 ± 17 months (median = 7). Seminal HIV shedding occurred in 13% of patients (46/362). No genito-urinary symptoms and/or infections were reported or diagnosed in any of the patients during follow-up. The blood virus load (NBVL) of about half the men (52%, *n* = 187) was always (626 samples) undetectable, it was either detectable or undetectable (PBVL + NBVL, 484 samples) for 22% of the men (*n* = 80) and always detectable (PBVL, 288 samples) for 26% of the men (*n* = 95). HIV seminal shedding was four times less frequent (5.3%) and at least 5 times less abundant (mean 213 HIV-1 RNA copies /ml [min <200 – max 4388]) in patients on effective ART than in patients with an uncontrolled blood HIV load (21.0% and 1146 HIV-1 RNA copies /ml [min <200 – max 308,500]).

### HIV seminal shedding

A total of 22 patients, the shedders, produced at least one NBVL + PSVL specimen during follow-up (6%, 22/362). This group represent 47% (22/46) of subjects with HIV shedding into the semen. These 22 shedders included 13 who always had an undetectable blood virus load and 9 who had at least one detectable blood virus load during follow-up. The members of this group all had similar recorded parameters and differed only in the duration of the HIV infection since diagnosis: 9 years for always undetectable shedders and 14 years for those whose blood HIV RNA was not always undetectable (*p* = 0.017). The mean seminal HIV load during isolated shedding episodes was 340 ± 613 RNA copies /ml (maximum 5600).

We compared the shedders (*n* = 22) with the controls, whose HIV loads in both compartments were always undetectable (NBVL + NSVL, *n* = 171) to identify factors associated with isolated HIV seminal shedding (Table 1).

**Table 1:** HIV infection, coinfection and treatment histories in patients in the 2 groups (*n* = 193 subjects)

	(IHS) At least once NBVL and PSVL *n* = 22		NBVL and NSVL *n* = 171	
	n	(%)	n	(%)
CD4 cell count (×10^6^/l)				
≤ 350	3	(14)	27	(16)
350–500	3	(14)	40	(24)
> 500	16	(72)	103	(60)
Mode of acquisition				
Sexual	11	(61)	98	(64)
IDU/blood	7	(39)	56	(36)
STI history	10	(53)	54	(35)
OI history	5	(25)	35	(22)
Co-infection HBV	2	(10)	13	(8)
Co-infection HCV	8	(40)	55	(34)
	Mean ± sd	(med)	Mean ± sd	(med)
Time since diagnosis (years)	11 ± 5	(12)	10 ± 6	(10)

*Abbreviations*: *IHS* Isolated HIV shedder; *NBVL* Negative Blood Virus Load; *PSVL* Positive Seminal Load; *NSVL* Negative Seminal Load; *IDU* Intravenous Drug User; *STI* Sexually Transmitted Infections; *OI* Opportunistic Infections; Mean ± sd for mean ± standard deviation, (med) for (median)

The mean ages of the patients in the two groups were similar: 41 ± 5 years (median = 42, min-max = 33–48) for shedders and 39 ± 7 (median = 39, min-max = 22–59) for controls. The average number of samples collected from individual shedders and controls differed significantly (*p* < 0.001). The average number of samples from shedders was 7 ± 5 (median = 6, min-max = 1–17), and the number from controls was 3 ± 2 (median = 3, min-max = 1–15).

The follow-up time for shedders, between the first and last samples collected, was 25 ± 25 months (median = 17, min-max = 0.13–95), and that for controls was 10 ± 14 months (median = 5, min-max = 0.16–78), *p* = 0.002.

We compared the HIV history parameters for shedders and controls: the CD4 cell counts, mode of transmission, STI history, opportunistic infections and co-infections with HBV and HCV (usually associated with intravenous drug use). None of these parameters was significantly different.

### Impact of HIV treatment on shedding

**Fig. 1:**
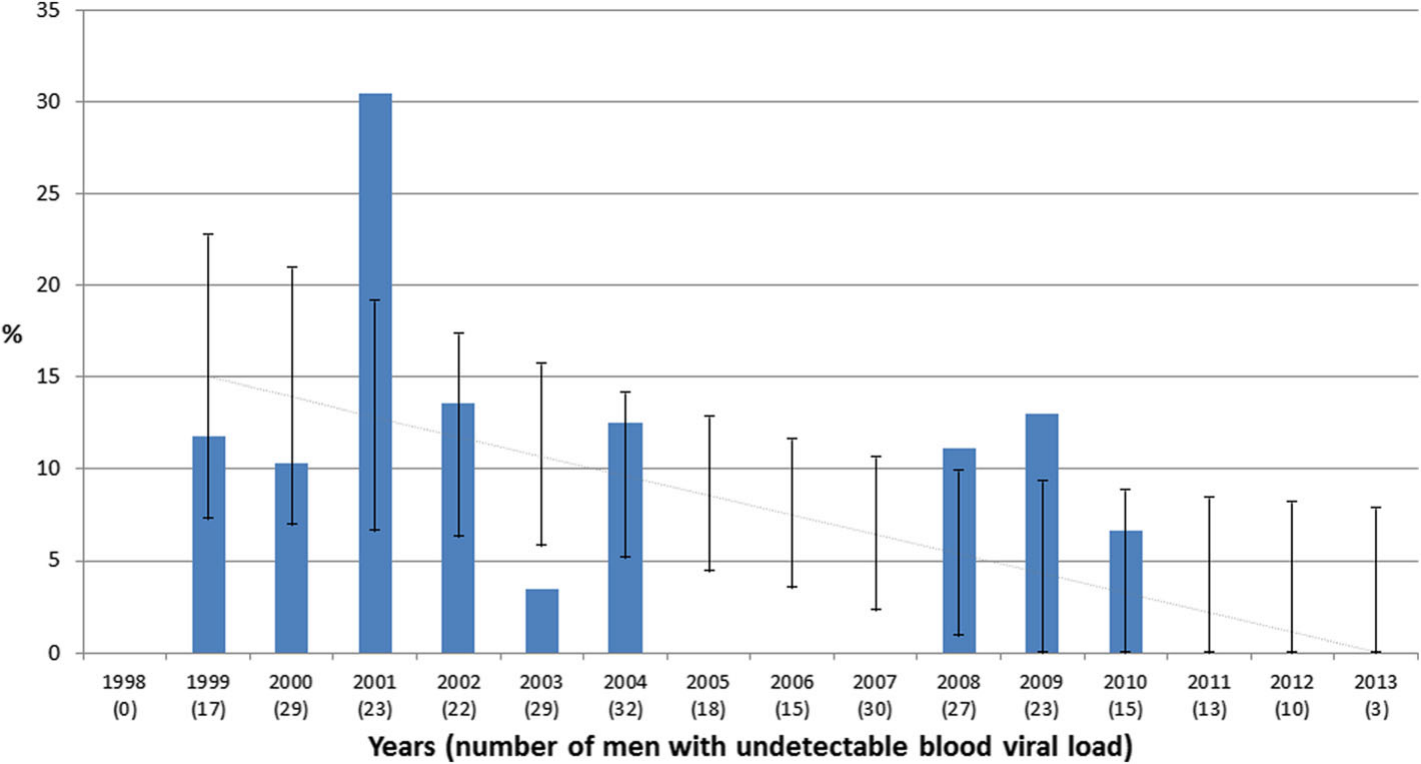
Evolution of the prevalence of patients with isolated HIV seminal shedding among patients with an undetectable blood virus load. Histogram (blue bars) shows the annual percentage of NBVL patients showing isolated HIV shedding (NBVL + PSVL) (numbers in brackets) during 1998–2013. The slope represents the estimated trend of the prevalence with associated confidence intervals. Abbreviations: PSVL (Positive Seminal Virus Load), NBVL (Negative Blood Virus Load)

We assessed the impact of antiretroviral treatment on HIV shedding into the semen (Table 2). The times between the first sample collected and the initiation of the first antiretroviral treatment were the same for the men in the two groups. But the duration of the current regimens during isolated HIV seminal shedding did differ: shedders were treated with their current regimen for longer than were the controls (*p* = 0.011). Shedders had been on more antiretroviral drug regimens (1.27) than the controls (1.06; *p* = 0.03). Nevertheless, the nature of the current antiretroviral combinations for the two groups did not differed significantly in terms of the number of drugs in each antiretroviral combination. The regimens of the two groups had similar NNRTI, PI or integrase inhibitors contents. The durations of the HIV infections in the 2 groups were also similar. Patients with an effective ART (NBVL) showed an overall reduction in the number of seminal HIV sheddings during the study period **(**Fig. 1 **)**.

**Table 2:** Antiretroviral regimens and treatment duration in the 2 groups (*n* = 193 subjects)

	(IHS) At least once NBVL and PSVL *n* = 22		NBVL and NSVL *n* = 171	
	Mean ± sd	(med)	Mean ± sd	(med)
Duration of treatment (years)	7 ± 3	6	7 ± 5	6
Duration of current treatment (years)$	3 ± 2	3	2 ± 2	2
	n	(%)	n	(%)
Current regimen				
Bi-therapy	2	(9)	10	(6)
Tri-multitherapy	20	(91)	161	(94)
NRTI				
None	0	(0)	6	(4)
One	1	(5)	8	(5)
≥ Two	21	(95)	157	(91)
NNRTI				
None	12	(55)	99	(58)
≥ One	10	(45)	72	(42)
PI				
None	12	(55)	92	(54)
≥ One	10	(45)	79	(46)
II				
None	22	(100)	169	(99)
One	0	(0)	2	(1)

*Abbreviations*: *IHS* Isolated HIV shedder; *NBVL* Negative Blood Virus Load; *PSVL* Positive Seminal Load; *NSVL* Negative Seminal Load; *NRTI* Nucleoside Reverse Transcriptase Inhibitor; *NNRTI* Non Nucleoside Reverse Transcriptase Inhibitor; *PI* Protease Inhibitor; *II* Integrase Inhibitor

$ *p* < 0.05 between group NBVL + PSVL (1) and group NBVL + NSVL (2)

### HIV shedding patterns

**Fig. 2:**
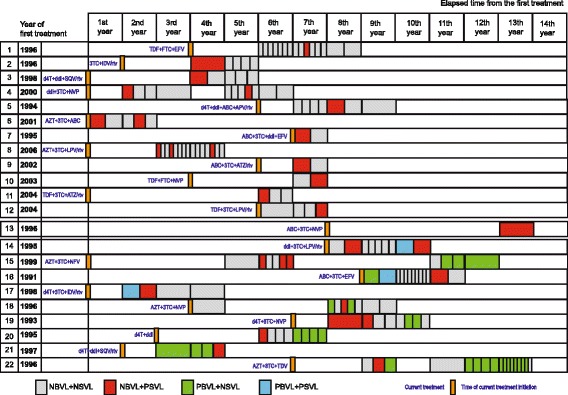
HIV shedding patterns of patients with isolated HIV seminal shedding. Diagram and chronological description of the paired blood and seminal virus loads for the 22 subjects who had at least once paired NBVL and PSVL (isolated HIV seminal shedding, shedders). The year when the first antiretroviral treatment of each subject was initiated is indicated in the second column and the years of follow-up after this date are numbered 1 to 14 on the first line. Orange bars indicate the initiation of the antiretroviral regimen used during follow-up and the antiretroviral drug combination is specified just before the orange bar. Each coloured case represents the result of one pair of blood and seminal viral loads: Grey cases indicate no HIV-1 RNA detected in either blood or seminal plasma (NBVL + NSVL), red cases indicate isolated seminal shedding (NBVL + PSVL), green cases indicate a blood plasma HIV-1 detected without seminal shedding (PBVL + NSVL) and blue cases indicate HIV-1 RNA detected in both blood and seminal plasma. Thus, patient 1 gave 15 pairs of samples during years 6 to 8 of ART, but only one of them was NBVL + PSVL. Abbreviations: 3TC (lamivudine), ABC (abacavir), ATZ (atazanavir), AZT (zidovudine), d4T (stavudine), ddI (didanosine), EFV (efavirenz), FTC (emticitabine), IDV (indinavir), LPV (lopinavir), NFV (nelfinavir), rtv (ritonavir), SQV (saquinavir), TDF (tenofovir). NSVL (Negative Seminal Virus Load), PSVL (Positive Seminal Virus Load), NBVL (Negative Blood Virus Load), PBVL (Positive Blood Virus Load)

The patterns of HIV shedding into the semen of the 22 shedders are illustrated in Fig. 2 **,** together with their blood HIV-1 RNA data. The four combinations of VL results (PBVL + PSVL, PBVL + NSVL, NBVL + PSVL and NBVL + NSVL) are shown for each sample date, as well as the starting dates of the first and current antiretroviral treatments. Only one of the 22 shedders (#13) had a single combination of paired blood and semen results, 12 had 2 combinations (#1–12), 6 had 3 combinations (#14–21) and one had all 4 combinations (#22). The isolated HIV seminal shedding (NBVL + PSVL) profile occurred within the first year of treatment and up to 13 years after starting the first antiretroviral treatment. The isolated HIV seminal shedding events occurred more than one year after starting the first treatment in all cases, except one (#6). The isolated HIV seminal shedding profile occurred within a year after changing the antiretroviral drug regimen in 3 patients (#6, #7, #14), but 1 to 5 years later for the others. Seminal HIV shedding was detected in 18 subjects who had been on antiretroviral treatment for more than 12 months and in 13 who had been treated for 24 months. However, 8 subjects (#1, #5, #10, #12, #14, #15, #18, #21, #22) showed no HIV seminal shedding (at least one sample with no HIV RNA detection in semen) before the isolated HIV seminal shedding episode, and none of them 8 ever had a PBVL during this period.

The maximum HIV-1 load in the seminal plasma during isolated HIV seminal shedding episodes was 5600 HIV RNA copies/ml and only two subjects had consecutive occurrences (#15 and #19).

We detected two patterns in patients with more than one pair of samples (#13 excluded). The first, most common, pattern was that of patients 1–12; they were always NBVL but their seminal virus loads differed (PSVL or NSVL). These patients had 1 to 12 NBVL and 1 to 3 PSVL. They are thus true NBVL + PSVL patients with an optimal blood response to ART. The median date of their first antiretroviral treatment was 2000. The second pattern, shown by 9 patients (#14–22), was at least one (*n* = 4) or more (2 in 2 cases, 3 in 1 case, 4 in 1 case and 13 in 1 case) PBVL episodes. Seminal HIV shedding occurred before, during and after episodes of PBVL and all these patients began their treatment before 2000 (median 1996).

## Discussion

The HIV RNA load in the seminal plasma was correlated with sexual transmission in patients without antiretroviral treatment but this could be questioned for patients under effective antiretroviral treatment. This retrospective study examines a large number of synchronized semen and blood samples taken over 16 years of follow-up of HIV-infected male patients attending our assisted reproduction centre. Residual HIV shedding occurred in 6.1% of the patients (22/362) on effective ART, or 6.6% if we include the two patients studied previously [18, 19]. This frequency of HIV shedding agrees well the findings of previous retrospective studies (3–6.6%) on patients attending reproductive centres [3, 8, 9, 11, 22] and is lower than that for men having sex with men (25%) [13] or other populations (30–48%) [5, 23]. Since the number of samples per patient was not controlled these percentage of HIV seminal shedding are only comparative between retrospective studies in a medically assisted procreation setting which is the case in a large majority of them. We find that isolated HIV shedding in the semen tends to become less frequent over time, which agrees with the findings of Dulioust et al. [3] but not with those of Lambert-Niclot et al. [10]. The improved efficacy and/or greater acceptability of treatment over the study period are probably better illustrated in longer studies. Confusing factors like breaks in compliance or pharmacological interference with antiretroviral efficacy may be involved, as illustrated in the HIV shedding patterns of patients 14–22. However this cannot be excluded for patients on effective ART despite the frequent measurements of HIV RNA in the blood during follow-up.

Our data do not reveal any links between the shedding of HIV into the semen and the patients’ histories of HIV infection, CD4 cell counts or their antiretroviral regimens, although they differed greatly. The only differences were in the number of regimen changes since the first treatment initiation and the duration of the on-going drug regimen. The subjects who underwent isolated HIV seminal shedding may, after a good initial compliance to treatment, have become weary of their ART. The shedding of HIV-1 via the semen by the shedders may then be linked to breaks in compliance that influence only the genital tract compartment because it is less accessible to one or more of the drugs used. Since it was a retrospective study other bias may be involved. The antiretroviral drugs do not all diffuse in the same way in the male genital tract [24, 25], but the effect of reduced compliance on shedding via the genital tract is unknown. We find that 9/22 shedders had suboptimal ART (BVL blips or persistent PBVL), probably because of breaks in treatment compliance. ARV treatment was not specifically monitored during this study but blood virus load is a most reliable indicator of adherence. It is unlikely that isolated HIV seminal shedding is linked to HIV resistance to ART because that would probably lead to continuous shedding with a regular increase in seminal HIV load, and there have been no reports of resistant HIV developing in cases of persistent, abundant seminal HIV shedding [18, 19].

We did not record data on the presence of markers of inflammation, like polymorphonuclear cells and round cells, pro-inflammatory cytokines, or microbiome, in the semen of our patients [17, 26–28]. Similarly, the semen samples were too small for us to retrospectively check for any concomitant asymptomatic seminal shedding of *Human cytomegalovirus* or *Human herpesvirus 2*, which are known to be associated with increased HIV shedding, but can have little or no influence on the blood virus load [14].

The seminal plasma virus load is considered to be a predictor of the risk of transmission that is independent of the plasma virus load in untreated patients [29]. There is no reason to suppose that this is not the case for HIV–treated patients except for regimens that include a protease inhibitor, leading to production of non-infectious HIV particles. The precise amount of HIV in the seminal plasma that results in HIV being transmitted to a woman partner is not known. The impact of isolated semen HIV shedding on the sexual transmission rate of HIV is still a question. It is thus most important to understand how and why these genital and blood virus loads differ. Both the frequency of isolated HIV seminal shedding events and the quantity of HIV shed may influence the sexual transmission of HIV. Only one sample from a shedder (1/30) contained more than 5000 HIV RNA copies /ml (i.e. 5600 copies/ml), which raises the question of the infectivity of the seminal virus. It may not be a simple question of the number of virus particles; the virus may be defective or immature (due to protease inhibitor drugs). Sexual transmission also depends on the partner’s sensitivity, associated STI and sexual practice. The two patients excluded from this study each had more than 100,000 copies /ml of SVL while viral blood load was suppressed for more than 6 months and the risk of sexual transmission seems obvious [18, 19]. The sexual transmission of HIV may also be mediated by HIV-infected cells; HIV DNA associated with cells has been detected in patients on effective ART [12, 17, 30]. To date, only one case of male to male sexual transmission, while the index case was under effective HAART has been reported and discussed [31, 32].

Because patients provided several semen samples during the follow-up we were able to carry out a longitudinal analysis of the concordance/discordance between the data for the blood and seminal compartments. Isolated seminal shedding can occur more than 6 months after the initial treatment began (21/22) for our patients, and over 1 year after initiation in most cases. Moreover, this study analyses the precise isolated HIV seminal shedding patterns according to the time of first ART initiation and initiation of the current regimen.

## Conclusions

This longitudinal study indicates that HIV-1 shedding was four times less frequent (5.3%) in patients on effective antiretroviral treatment (< 20 HIV-1 RNA copies /ml blood plasma) than in the total population of antiretroviral-treated patients studied. Isolated HIV shedding can occur regardless of the ART regimen and even when the HIV blood viral load is blocked for up to 5 years after effective ART initiation. The HIV seminal shedding patterns were probably due to breaks in treatment compliance or associated with local factors specific to the genital compartment, although breaks in treatment compliance that influence only the genital compartment cannot be excluded. Detection of HIV RNA in the semen was the gold standard for evaluating the risk of sexual HIV transmission. This is now in question because sexual transmission by a patient on effective ART has not yet been reported.

We believe that HIV RNA should be assayed in the semen both in cases in which the infected partner recently started ART (within 6 months) and afterward, particularly if he had history of poor adherence to ART.

## Abbreviations

ART: Anti-Retroviral Therapy
BVL: Blood Virus Load
NBVL: Negative Blood Virus Load
NSVL: Negative Seminal Virus Load
PBVL: Positive Blood Virus Load
PSVL: Positive Seminal Virus Load
STI: Sexually Transmitted Infections
SVL: Seminal Virus Load
